# The *Listeria monocytogenes* Bile Stimulon under Acidic Conditions Is Characterized by Strain-Specific Patterns and the Upregulation of Motility, Cell Wall Modification Functions, and the PrfA Regulon

**DOI:** 10.3389/fmicb.2018.00120

**Published:** 2018-02-06

**Authors:** Veronica Guariglia-Oropeza, Renato H. Orsi, Claudia Guldimann, Martin Wiedmann, Kathryn J. Boor

**Affiliations:** Food Safety Laboratory, Department of Food Science, Cornell University, Ithaca, NY, United States

**Keywords:** RNA-seq, *Listeria monocytogenes*, lineages, sigB, PrfA

## Abstract

*Listeria monocytogenes* uses a variety of transcriptional regulation strategies to adapt to the extra-host environment, the gastrointestinal tract, and the intracellular host environment. While the alternative sigma factor SigB has been proposed to be a key transcriptional regulator that facilitates *L. monocytogenes* adaptation to the gastrointestinal environment, the *L. monocytogenes*' transcriptional response to bile exposure is not well-understood. RNA-seq characterization of the bile stimulon was performed in two *L. monocytogenes* strains representing lineages I and II. Exposure to bile at pH 5.5 elicited a large transcriptomic response with ~16 and 23% of genes showing differential transcription in 10403S and H7858, respectively. The bile stimulon includes genes involved in motility and cell wall modification mechanisms, as well as genes in the PrfA regulon, which likely facilitate survival during the gastrointestinal stages of infection that follow bile exposure. The fact that bile exposure induced the PrfA regulon, but did not induce further upregulation of the SigB regulon (beyond that expected by exposure to pH 5.5), suggests a model where at the earlier stages of gastrointestinal infection (e.g., acid exposure in the stomach), SigB-dependent gene expression plays an important role. Subsequent exposure to bile induces the PrfA regulon, potentially priming *L. monocytogenes* for subsequent intracellular infection stages. Some members of the bile stimulon showed lineage- or strain-specific distribution when 27 *Listeria* genomes were analyzed. Even though *sigB* null mutants showed increased sensitivity to bile, the SigB regulon was not found to be upregulated in response to bile beyond levels expected by exposure to pH 5.5. Comparison of wildtype and corresponding Δ*sigB* strains newly identified 26 SigB-dependent genes, all with upstream putative SigB-dependent promoters.

## Introduction

*Listeria monocytogenes* is a ubiquitous Gram-positive bacterium that can cause a life-threatening, invasive disease called listeriosis in humans and animals. *L. monocytogenes* is the third most common cause of death from foodborne infections in the USA (Scallan et al., 2011) and has been estimated to cause over 23,000 cases of listeriosis, 23.6% of them resulting in death, each year worldwide (Maertens de Noordhout et al., 2014). The genus *Listeria* includes 17 species, two of which, *L. monocytogenes* and *L. ivanovii*, are considered pathogenic; *L. monocytogenes* has been associated with human and animal infections, while *L. ivanovii* has primarily been associated with animal infections (Orsi and Wiedmann, 2016). Within *L. monocytogenes*, four evolutionary distinct lineages (I, II, III, and IV) have been established. Most isolates, however, are grouped within lineage I or lineage II (Orsi et al., 2011). Lineage I and lineage II comprise the serotypes more commonly associated with human clinical cases, serotypes 1/2b and 4b and serotype 1/2a, respectively. In most countries and regions, lineage I is overrepresented among human isolates, while lineage II is over-represented among food isolates (Jeffers et al., 2001; Gray et al., 2004; Manuel et al., 2015), suggesting that these lineages may differ in their ecological niches and hence their adaptive responses to different stress conditions (Orsi et al., 2011).

*L. monocytogenes* is characterized by its ability to survive and thrive under different and rapidly changing environmental conditions. Some of the stressful conditions encountered by *L. monocytogenes* include those encountered in the extra-host environment, as well as in those associated with the extra- and intra- cellular environment of the host. Common stresses that *L. monocytogenes* is exposed to include the rapidly changing conditions encountered during passage through the gastrointestinal (GI) tract (Nicaogain and O'Byrne, 2016). Crucial to this bacterial resilience is the ability to modify the appropriate regulatory networks in response to environmental cues. A key regulator of *L. monocytogenes*' resilience is the alternative sigma factor, SigB. SigB has been shown to regulate over 200 genes with important roles in virulence and stress response (e.g., acid resistance, osmotic stress resistance, arsenate resistance, oxidative stress resistance, cold stress and transition to the stationary growth phase) with the majority of studies conducted using lineage II strains (mainly 10403S and EGD-e) (Severino et al., 2007; Hain et al., 2008; O'Byrne and Karatzas, 2008; Raengpradub et al., 2008; Mujahid et al., 2013).

As infection with *L. monocytogenes* progresses beyond the GI tract, the onset of a successful invasive disease is facilitated by another master regulator, PrfA. PrfA regulates 19 genes involved in virulence, including the bile salt hydrolase *bsh* (Scortti et al., 2007; Freitag et al., 2009; de las Heras et al., 2011; Vasanthakrishnan et al., 2015). PrfA itself is tightly regulated, and SigB has been shown to play a well-established positive role in *prfA* transcription (Kazmierczak et al., 2006; Ollinger et al., 2009) as well as a less-understood, indirect role in the post-transcriptional downregulation of PrfA (Ollinger et al., 2009).

Bile is one of the stresses *L. monocytogenes* encounters in the upper intestinal tract. While bile can be harmful to non-commensal bacteria due to its detergent-like antimicrobial activity, it has been hypothesized that bile could also be sensed by bacteria as an environmental signal (Dowd et al., 2011; Sistrunk et al., 2016). Under normal physiological conditions, the bile concentration in the human gut is between 0.005 and 2% (Dawson, 1998). Strikingly, *L. monocytogenes* is able to grow in gall bladders (Hardy et al., 2004, 2006; Huff et al., 2005; Jensen et al., 2008; Dowd et al., 2011), where bile is concentrated at neutral pH. However, once bile is released into the duodenum postprandially, it becomes active in this low pH environment (Dowd et al., 2011). Previous proteomic and transcriptomic studies of the *L. monocytogenes* bile stimulon have been performed under aerobic and anaerobic conditions but at neutral pH (Quillin et al., 2011; Payne et al., 2013; Wright et al., 2016). To better understand the response of *L. monocytogenes* to conditions encountered in the proximal part of the small intestine, we characterized the transcriptomic response triggered by exposure to 1.1% bile in an acidic (pH 5.5) environment in two strains of *L. monocytogenes* representing lineages I and II.

## Materials and methods

### Bacterial strains

Two well-characterized *L. monocytogenes* strains were used for the experiments reported here, including strain H7858 (lineage I, serotype 4b) and 10403S (lineage II, serotype 1/2a, ST85) (Table 1). Although H7858 itself has not been sequence typed, nine other serotype 4b isolates associated with the same 1998 U.S. multistate hot dog outbreak all have been sequence typed as ST6 (Chen et al., 2016), strongly suggesting that H7858 is ST6. Strains representing serotypes 1/2a (lineage II) and 4b (lineage I) were chosen as these two represent the most commonly isolated *L. monocytogenes* serotypes from foods and human clinical specimens, respectively (Orsi et al., 2011). The specific strains used here were selected as high quality genome sequences and previously characterized corresponding isogenic *sigB* mutants were available for both strains (Wiedmann et al., 1998; Ferreira et al., 2001; Bergholz et al., 2013).

**Table 1:** Strains used in this study.

**Lineage**	**Strain**	**Genotype**	**Origin**	**Identification**	**References**
I	H7858	WT	1998 hot dog outbrea0^*^k, US	FSL F6-0366	Nelson et al., 2004
I	H7858	Δ*sigB*	H7858	FSL K5-0018	Bergholz et al., 2013
II	10403S	WT	Laboratory type strain	FSL X1-0001	Bishop and Hinrichs, 1987
II	10403S	Δ*sigB*	10403S	FSL A1-0254	Wiedmann et al., 1998

### Growth conditions

Both parent strains and their isogenic *sigB* mutants were maintained in glycerol stocks at −80°C. Strains were streaked from 15% glycerol stocks on BHI agar, and plates were incubated for 16 h at 37°C. A fresh single colony from these plates was inoculated into 5 mL tubes of BHI broth, which then were subsequently incubated at 37°C for 16 h (with shaking). The resulting overnight cultures were diluted 1/100 into 100 ml of BHI broth (in flasks), followed by incubation at 37°C (without shaking) for 3 h to allow cultures to reach mid log phase (defined as OD_600_ of ~0.4; 3 to 5 x10^8^ cfu/ml). Once mid log phase was reached, cells were diluted 1/10 into 90 ml pH 5.5 BHI broth, pre-warmed at 37°C, which contained 1.1% porcine bile (Sigma, St. Louis, MO). Controls were diluted into pH 5.5 BHI broth, pre-warmed at 37°C, without bile. Assay and control flasks were returned to 37°C. For bile sensitivity assays, samples were collected right before dilution into bile media, and 0, 10, or 20 min after exposure to bile. At each time point, samples were serially diluted into sterile PBS and plated using an automatic spiral plater (AutoPlate—Spiral Biotech, Inc., Norwood, MA). CFU/mL were calculated from plates using a Q-count plate reader (Spiral Biotech, Inc.).

### RNA isolation

To assess the *L. monocytogenes* response to bile stress (1.1% bile in BHI adjusted to pH 5.5), cells were collected for RNA extraction after 10 min of bile exposure; all treatments were conducted in four biological replicates conducted on different days. For each strain and condition, 300 ml of cells were collected in three separate centrifugation tubes (100 ml/tube). RNA was subsequently extracted using a modified Phenol-Chloroform extraction protocol (Tang et al., 2015). Briefly, bacterial growth was halted with 1% final volume phenol:ethanol, followed by chilling of the suspension and collection of cell pellets by centrifugation at 4°C. Pellets were then resuspended in 100 μl of TE containing 15 mg/ml lysozyme, followed by addition of 20 μl of 20 mg/ml proteinase K and incubation at 37°C for 30 min. Cell lysates were subsequently mixed with 1 ml of Tri Reagent (Ambion, Austin, TX), transferred into bead-beating tubes that contained 3 ml of 0.1 mm zirconium beads, followed by adjustment (with Tri Reagent) to a final volume of 5 ml. The three lysates from the same treatment were pooled at this stage and processed in the bead-beater at maximum speed for 4 min. After bead-beating, samples were centrifuged for 10 min at 4,637 × g, 4°C. Supernatants were separated from the beads, transferred into a new tube, and mixed with 500 μl of bromo-chloro-propane (BCP). After incubation with BCP for 10 min, samples were centrifuged at 14,637 × g for 15 min at 4°C. The aqueous layer was collected and nucleic acids were precipitated overnight at −80°C using 2.5 volumes of 100% ice-cold isopropanol. Nucleic acids were pelleted, washed with 75% ethanol, and resuspended in 100 μL nuclease free water. DNA was removed from samples using Turbo DNAse (Ambion), followed by phenol-chloroform extraction and RNA precipitation. After pelleting and washing with 70% ethanol, RNA was resuspended in 100 μl nuclease free water. Total RNA concentration and quality were assessed using a Nanodrop (ThermoScientific, South San Francisco, CA) and a Nano chip run on a 2100 Bioanalyzer (Agilent Technology, Santa Clara, CA).

### rRNA depletion, library preparation, and sequencing

RNA collected from four independent biological replicas, performed on different days, was used for cDNA library preparation. rRNA depletion and construction of directional cDNA libraries were performed using the ScriptSeq Complete Kit (Bacteria)-Low Input (Epicenter, Madison, WI). As a first step, rRNA depletion was performed with Ribo-Zero rRNA Removal Reagents (Bacteria)-Low Input and the Magnetic Core Kit-Low Input. rRNA-depleted samples were checked using a Pico chip run on a 2100 Bioanalyzer (Agilent Technology), to confirm depletion of 16S and 23S rRNA, and then purified using the Agencourt RNAClean XP Kit (Beckman Coulter Inc., Brea, CA). cDNA libraries were prepared and indexed following the ScriptSeq™ v2 RNA-seq Library Preparation Procedure. The quality of cDNA libraries was assessed with a High-Sensitivity DNA chip run on a 2100 Bioanalyzer (Agilent Technology), as recommended by the manufacturer. Indexed RNA-seq libraries were quantified by digital PCR, and sequencing was carried out on a Hiseq 2500 (single-end, 150-bp per read) at the Cornell Core Facility for RNA-sequencing. RNA-seq data have been deposited in NCBI's Gene Expression Omnibus and are accessible through GEO Series accession number GSE103443.

### RNA-seq analysis

Sequence reads originated from experiments using strains 10403S and H7858 were aligned to a 10403S finished genome or an H7858 high quality unfinished pseudochromosome (Tang et al., 2015), respectively, using the BWA mem algorithm in BWA version 0.7.3a (Li and Durbin, 2010) and the data for coverage per base on sense and antisense strands were analyzed separately using samtools (Li et al., 2009). On average, for 10403S, 94% of the reads mapped to the corresponding genome; and for H7858, 93% of the reads mapped to the genome.

Differential expression (DE) of protein-coding genes and non-coding RNA (ncRNAs) features was analyzed using the Bayseq package for R version 2.2.0 (Hardcastle and Kelly, 2010) based on the total coverage obtained for each gene or feature. Six different DE analyses were performed to compare transcript levels between (i) strain 10403S with and without exposure to bile; (ii) strain H7858 with and without exposure to bile; (iii) 10403S parent and Δ*sigB* strain both not exposed to bile; (iv) 10403S parent and Δ*sigB* strain both exposed to bile; (v) H7858 parent and Δ*sigB* strain both not exposed to bile and (vi) H7858 parent and Δ*sigB* strain both exposed to bile. For each comparison, we created an NDE model for Non Differentially Expressed and a DE model for Differentially Expressed. Likelihoods that a gene or feature belongs to the DE model and their respective False Discovery Rate (FDR) were estimated. Genes or features with FDR values < 0.05 and Fold Change (FC) of either < 0.4 or > 2.5 (representing up- or down-regulation of >2.5-fold) were considered significantly differentially expressed. Since RNA-seq has been shown in *L. monocytogenes* (Oliver et al., 2009) and other organisms (Griffith et al., 2010; Vivancos et al., 2010; Wu et al., 2014) to provide quantitative data that are well correlated with qPCR data, qPCR confirmation of genes identified here was not considered necessary.

### Gene set enrichment analysis

The GOseq 1.18.0 package for R (Young et al., 2010) was used as previously described (Tang et al., 2015) to assess whether differentially transcribed genes (i.e., genes classified into the DE model) were enriched for Gene Ontology (GO) terms. GO term annotations for 10403S and H7858 were combined into a single GO term annotation to ensure that genes from both strains were assessed with the same GO terms; this combined dataset is available on GitHub (https://github.com/renatorsi/Guariglia-Oropeza2017BileStimulonL_monocytogenes).

### Identification and characterization of bile-responsive and SigB-dependent genes in additional *L. monocytogenes* and *Listeria* spp. genomes

A set of 27 finished genomes representing 10 *L. monocytogenes* lineage I strains, 9 *L. monocytogenes* lineage II strains, 4 *L. monocytogenes* lineage III strains, 1 *Listeria innocua* strain, 1 *Listeria seeligeri* strain, 1 *Listeria ivanovii* strain and 1 *Listeria welshimeri* strain (Liu et al., 2016) was used to assess whether genes found to be 10403S- or H7858-specific were present or absent from other *Listeria* genomes. BlastN (Altschul et al., 1990), using the nucleotide sequence of 10403S or H7858 genes as queries, was used against a database containing the 27 finished genomes. These 27 genomes were the only closed genomes available at the time; we only used closed genomes to reduce the risk of misclassifying a gene as absent due to regions not sequenced in a non-closed genome. Matches with coverage >70% and identity >60% were considered relevant and the gene was assigned as “present” in the respective genome. Matches below the coverage or identity thresholds were assigned as “absent” in the respective genome.

### Association analysis between bile-responsive genes and the SigB or the PrfA regulon

A gene set representing the SigB regulon (241 genes) was compiled to include several studies and genes with previously annotated SigB promoters (Liu et al., 2017). A gene set representing the PrfA regulon (19 genes) was compiled to include several studies and genes with previously annotated PrfA boxes (Milohanic et al., 2003; Scortti et al., 2007; Ollinger et al., 2008, 2009). Both of these gene sets were used to assess how bile exposure affected transcript levels of the SigB and PrfA regulons, and are available on GitHub (https://github.com/renatorsi/Guariglia-Oropeza2017BileStimulonL_monocytogenes). For each strain, two-sided Fisher's exact tests were used to assess whether (i) genes up-regulated in bile were associated with SigB regulon, (ii) genes down-regulated in bile were associated with SigB regulon, (iii) genes up-regulated in bile were associated with PrfA regulon and (iv) genes down-regulated in bile were associated with PrfA regulon. FDR correction for multiple testing was applied and adjusted *p* < 0.05 were considered significant.

## Results

### *L. monocytogenes* transcriptional response to bile under acidic conditions includes differential regulation of >15% of *L. monocytogenes* genes

**Figure 1:**
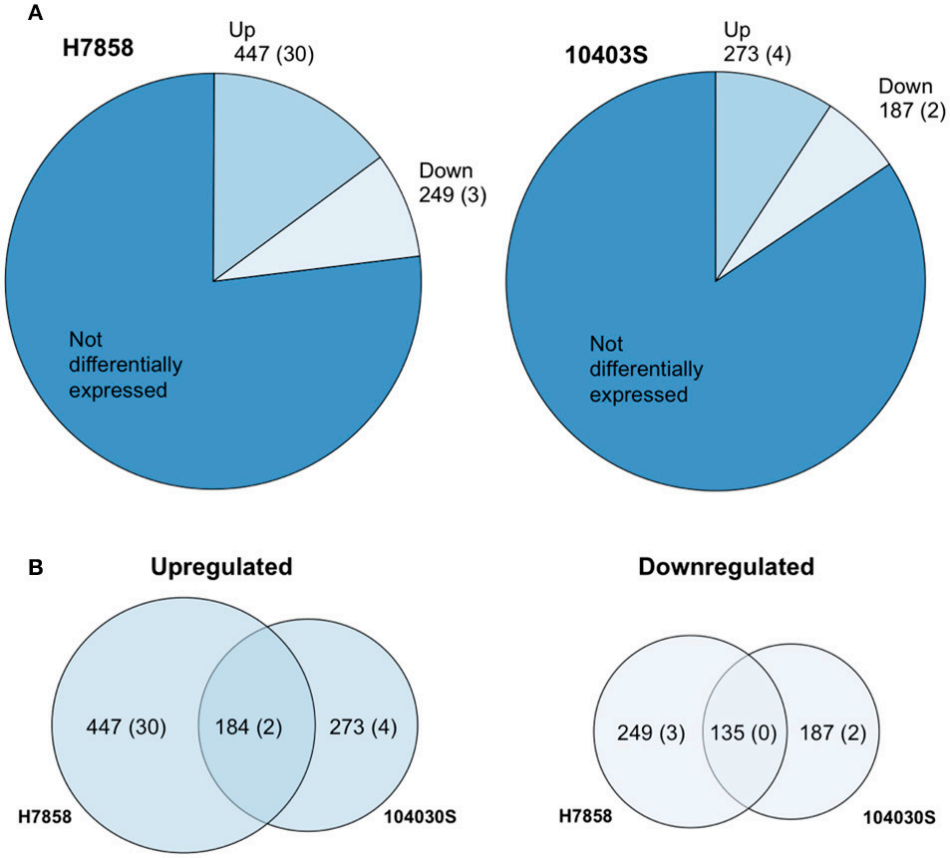
The Bile Stimulon. **(A)** Pie charts showing the number of *L. monocytogenes* total transcripts found to be affected by exposure to bile at pH 5.5 in strains H7858 and 10403S, with number of ncRNAs in parenthesis. H7858 and 10403S genomes include, respectively, a total of 3029 and 2956 annotated genes and ncRNAs. **(B)** Proportional Venn diagram showing the overlap of transcripts with significantly higher (Upregulated) or lower (Downregulated) levels after exposure to bile in H7858 (left) and 10403S (right), with number of non-coding RNAs in parenthesis.

We selected a serotype 1/2a strain to represent lineage II and a serotype 4b strain to represent lineage I as these serotypes are the most commonly isolated serotypes from food or environmental sources, and from human listeriosis outbreaks, respectively. To define the bile response for *L. monocytogenes* strains 10403S (Lineage II, serotype 1/2a) and H7858 (Lineage I, 4b), we used RNA-seq data for both strains exposed to pH 5.5 with and without bile for 10 min. This time point was chosen since a reduction in CFU/ml for both parent strains and their isogenic Δ*sigB* mutant strains was already observed after 10 min of bile exposure (Supplementary Figure 1). Genes with False Discovery Rate (FDR) values < 0.05 and Fold Change (FC) either < 0.4 or >2.5 (representing up- or down-regulation of >2.5-fold) were considered significant. For strain 10403S, ~16% of annotated protein-coding genes and non-protein-coding RNAs (ncRNAs) (460/2956) were identified as showing differential transcript levels (either up- or downregulation) after bile exposure for 10 min. For strain H7858, 23% of annotated protein-coding genes and ncRNAs (696/3029) showed differential transcript levels (Figure 1, Supplementary Table 1).

For strain 10403S, 269 protein-coding genes and 4 ncRNAs showed higher transcript levels in the bile-exposed bacteria as compared to 417 protein-coding genes and 30 ncRNAs in H7858. Combined analysis of the data for both strains found a bile pan-stimulon (defined as genes that showed significantly higher transcript levels after bile exposure in at least one strain) of 504 protein-coding genes and 32 ncRNAs. The core-stimulon (defined as genes that showed significantly higher transcript levels after bile exposure in both 10403S and H7858) included 182 protein-coding genes and 2 ncRNAs (Figure 1, Supplementary Table 1).

In addition to the genes upregulated after bile exposure, we also found 185 protein-coding genes and 2 ncRNAs that showed lower transcript levels after bile exposure in strain 10403S as compared to 246 protein-coding genes and 3 ncRNAs in H7858. Among these protein-coding genes, 135 showed lower transcript levels after bile exposure in both strains; no ncRNAs showed lower transcript levels in both strains (Figure 1, Supplementary Table 1).

### The bile stimulon is characterized by a broad response that includes upregulation of motility and cell wall modification genes as well as the PrfA regulon

To further characterize the transcriptional response to bile, we performed a GO term enrichment analysis of the protein-coding genes that showed higher transcript levels after bile exposure. This analysis identified 11 and 47 GO terms that are overrepresented among upregulated genes in 10403S and H7858, respectively (Supplementary Table 2). The 6 GO terms that were common among both strains included three different GO terms associated with transport functions (e.g., “transporter activity”) as well as GO terms annotated as (i) signal transduction, (ii) phosphoenolpyruvate-dependent sugar phosphotransferase system, and (iii) cell communication (Supplementary Table 2). In addition, GO terms associated with cell motility and locomotion were enriched in both strains, even though the specific GO terms enriched in 10403S (“cell motility”) and in H7858 (e.g., “chemotaxis,” “locomotion”) differed (Supplementary Table 2).

**Figure 2:**
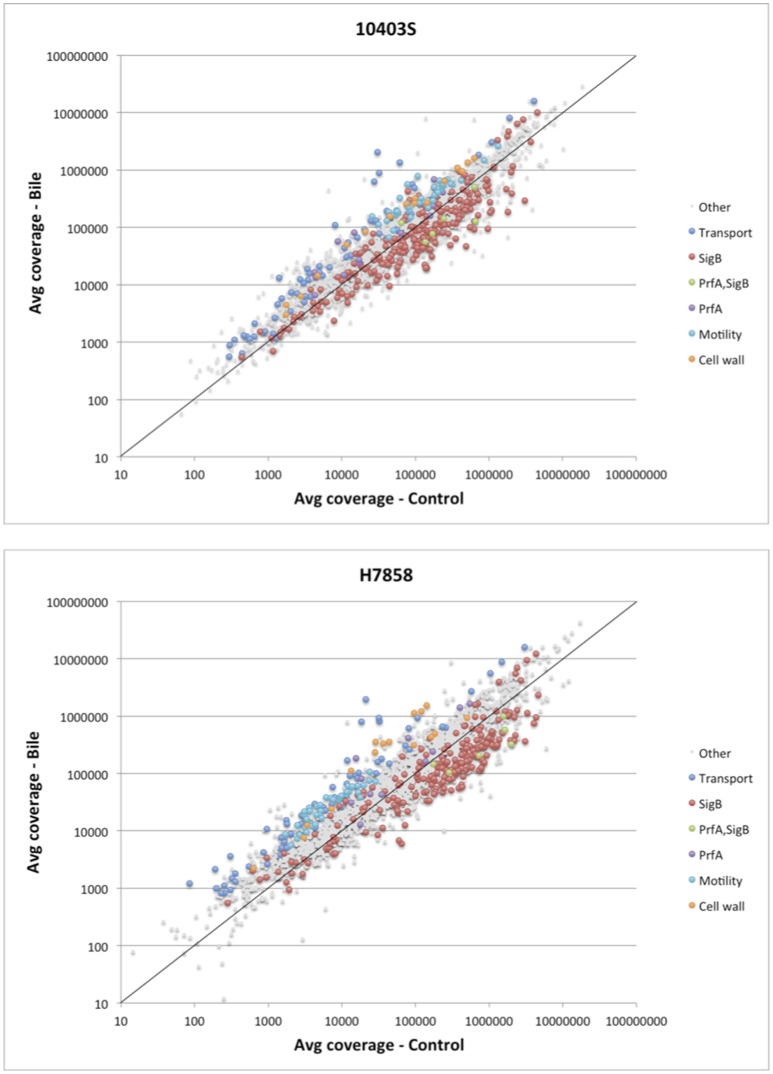
Analysis of differential expression on the RNA-seq data of *L. monocytogenes* exposed to pH 5.5 with and without bile. Scatterplot representing the average expression level of genes exposed to pH 5.5 with bile (Y axis) vs. without bile (X axis) in *L. monocytogenes* 10403S (Top) and H7858 (Bottom). The known regulons of SigB and PrfA are labeled, as well as the significantly upregulated genes belonging to transport, cell wall, and motility related functions. The x = y line is shown in solid black, indicating a ratio of one (non-differentially expressed).

Consistent with enrichment of “cell motility” and “locomotion” GO terms, six transcriptional units encoding flagellar and motility related functions showed higher transcript levels after bile exposure in either both 10403S and H7858 or in only H7858 (Figure 2, Supplementary Table 3). These upregulated genes included the chemotactic response genes *cheA* and *cheY* (found to be upregulated in both strains), as well as several genes encoding flagellar proteins. Interestingly, most of the flagellar motility genes that were significantly upregulated in H7858, also had higher transcripts in 10403S exposed to bile, but did not meet the FC threshold of >2.5 (although most of these genes had a FC>1.5) (Supplementary Table 3).

Further review of individual genes and operons that showed significantly higher transcript levels under bile exposure compared to the control conditions identified a number of genes encoding proteins involved in cell wall modification as well as some genes likely involved in homeostasis pathways (Figure 2, Supplementary Table 3). For example, the *dltABCD* operon showed higher transcript levels under bile exposure compared to the control with an average FC between all genes in the operon of 2.9 and 10.8 for strains 10403S and H7858, respectively. Additionally, several other genes encoding proteins with cell wall modification and homeostasis functions were found induced by bile in both 10403S and H7858, such as an operon involved in cell division and Mg transport (LMRG_02233 – LMRG_02235; peg_2495-peg_2497), an operon encoding cell surface proteins (LMRG_01646-LMRG_01647; peg_2052-peg_2053), and genes encoding N-Acetylmuramoyl-L-alanine amidases (LMRG_02131 and LMRG_02238; peg_2885 and peg_2499) (Supplementary Table 3).

**Figure 3:**
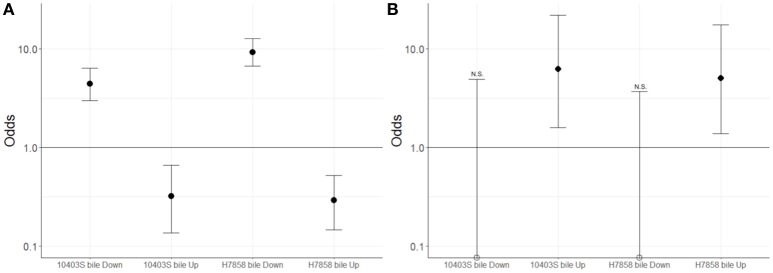
Association between genes classified here into the bile stimulon and SigB or PrfA dependent genes. For strains 10403S and H7858, two-sided Fisher's exact test was used to assess whether genes up- or down-regulated in bile were associated with **(A)** the SigB regulon or **(B)** the PrfA regulon. Odds ratio values are plotted with error bars representing the 95% confidence intervals. The y-axis is shown in log scale. FDR correction for multiple testing was applied and adjusted *p* < 0.05 were considered significant. Empty circles represent odds ratio values that were exactly zero and were therefore plotted on the x-axis. N.S. indicates odds ratio values that are not significantly different from 1; all odds ratio values that are not marked with NS were found to be significantly different from 1.

Since we hypothesized that expression of the SigB and PrfA regulons would be affected by bile, we determined whether genes classified into either the PrfA or SigB regulon were over- or underrepresented among the genes significantly upregulated under bile exposure. Separate analyses for both the 10403S and H7858 showed that in each of these two strains the PrfA regulon was overrepresented among the genes upregulated by bile (adjusted *p* = 0.018 and adjusted *p* = 0.027, respectively) (Figure 3). Some of the PrfA regulon genes induced under bile exposure included *hly, mpl, actA, plcA*, and *plcB* (Supplementary Table 3), whereas *inlA, inlB* and *prfA* itself, known PrfA regulon members that are also regulated by SigB, were not found to be upregulated under bile exposure. Genes classified into the SigB regulon were underrepresented among genes upregulated by bile in both 10403S and H7858 (adjusted *p*-values of 0.002 and < 0.001, respectively) (Figure 3).

### The bile response includes downregulation of oxidation-reduction processes as well as the SigB regulon

A GO term enrichment analysis performed with the genes that showed lower transcript levels after exposure to bile at pH 5.5 compared to non-exposed at pH 5.5 identified 22 GO terms enriched in 10403S and 13 in H7858; of these, 10 were common (Supplementary Table 4). Several oxidation-reduction processes terms were enriched in the genes downregulated by bile in both 10403S and H7858, including GO terms for oxidoreductase activity and antioxidant activity (Supplementary Table 4).

Additionally, genes classified as part of the SigB regulon were significantly overrepresented among the genes with lower transcript levels after exposure to bile in both 10403S and H7858 (adjusted *p* < 0.001 for both) (Figure 3). Consistent with this finding, four GO terms previously identified as enriched among SigB-regulated genes [GO:0015418, GO:0015697, GO:0030104, and GO:0015695; see Supplementary Table 4 (Liu et al., 2017)] were also identified here as significantly overrepresented among the genes with lower transcript levels after exposure to bile. SigB-dependent genes with lower transcript levels after bile exposure included *inlA* and *inlB*, which are not only SigB-regulated but also part of the PrfA regulon.

To characterize SigB-dependent and independent transcription patterns in *L. monocytogenes* exposed to bile, we also compared the effect of exposure to bile on the transcript levels of the 10403S and H7858 Δ*sigB* mutants. As predicted, we found that most GO terms previously found to be associated with SigB-regulated genes were not over-represented among the genes downregulated after bile exposure on the Δ*sigB* strains (see Supplementary Table 4). However, most GO terms for oxidation-reduction processes were still enriched among the genes downregulated after bile exposure on the Δ*sigB* strains, supporting SigB-independent downregulation of oxidation-reduction processes after bile exposure.

### The bile stimulon includes a number of lineage I and lineage II specific genes

Since strains 10403S and H7858 are classified into lineages II and I, respectively, we also used our RNA-seq data and subsequent comparative genomics analyses to identify strain- and lineage- specific members of the bile pan-stimulon. We first identified genes that showed significantly higher or lower transcript levels after bile exposure in one strain, but with no homologs identified in the genome sequence of the other strains. Subsequent BLAST searches were performed to characterize the distribution of these genes among closed genomes representing *L. monocytogenes* lineages I, II, and III (*n* = 10, 9, and 4, respectively) as well as other *Listeria* species (*L. innocua, L. ivanovii, L. seeligeri*, and *L. welshimeri*).

Among the 273 genes identified in the RNA-seq data analysis as upregulated under bile exposure in the lineage II strain 10403S, 20 were identified as having no homologs in the lineage I strain H7858 (Table 2). These 20 genes included (i) 9 genes present in all lineage II strains and absent from all lineage I strains, and (ii) 11 genes present in some, but not all, lineage II strains. Among the genes present in all lineage II strains, six were classified as “lineage II-specific” (meaning they were not found in any non-lineage II genomes) (see Table 2 for details); five of these genes (LMRG_01117—LMRG_01121) are part of an operon that includes genes encoding an integral membrane protein, a PTS system enzyme and a GntR family transcriptional regulator.

**Table 2:** Genes upregulated by bile in 10403S with no homolog in H7858.

**10403S Locus (LMRG_)**	**FC**	**Function**	**Presence in^a^:**						
			**Lineage I**	**Lineage II**	**Lineage III**	***L. innocua***	***L. ivanovii***	***L. seeligeri***	***L. welshimeri***
01117	3.3	Hypothetical protein	–	+	–	–	–	–	–
01118	3.1	Integral membrane protein	–	+	–	–	–	–	–
01119	2.9	Pentitol PTS system enzyme II B component	–	+	–	–	–	–	–
01120	3.7	Lmo1973 protein	–	+	–	–	–	–	–
01121	3.1	Transcriptional regulator, GntR family	–	+	–	–	–	–	–
02232	6.0	Hypothetical protein	–	+	–	–	–	–	–
00423	4.0	D-allulose-6-phosphate 3-epimerase	–	+	–	–	+	–	+
00425	4.4	Lmo0737 protein	–	+	–	–	+	–	–
00523	2.5	HAMP domain protein	–	+	–	–	–	–	+
02135	4.6	CRISPR-associated protein, SAG0894 family	V^b^	V^c^	–	+	–	–	–
02136	5.1	CRISPR-associated protein, SAG0894 family	V^b^	V^c^	–	–	–	–	–
02137	4.7	CRISPR-associated protein, SAG0894 family	V^b^	V^c^	–	–	–	–	–
02138	7.3	CRISPR-associated protein, SAG0894 family	V^b^	V^c^	–	+	–	–	–
02908	4.3	Hypothetical protein	V^b^	V^c^	–	+	–	–	+
00151	5.0	Hypothetical protein	–	V^d^	–	–	–	–	–
00152	2.8	Hypothetical protein	–	V^d^	–	–	–	–	–
01555	3.3	Polypeptide ORF26	–	V^f^	–	–	–	–	–
01556	3.5	ORF27	–	V^f^	–	–	–	–	–
01514	3.6	Repressor (CI-like) [Bacteriophage A118]	–	V^e^	V^g^	+	–	–	–
01513	2.6	Hypothetical protein	–	V^h^	–	–	–	–	–

a *Gene distribution was characterized among 27 genomes representing L. monocytogenes lineage I (n = 10), lineage II (n = 9), lineage III (n = 4), as well as other Listeria species (L. innocua, L. ivanovii, L. seeligeri, and L. welshimeri; n = 1 each). Genes absent in all genomes searched are labeled as “– “, genes present in all genomes searched are labeled as “+” and genes that showed variable presence are labeled as “V”*.

b Present in SLCC2540 (3b), SLCC2755 (1/2b), SLCC2482 (7).

c Present in SLCC5850 and 085923 (1/2a).

d Present in SLCC5850 and 085923 (1/2a) and SLCC7179 (3a).

e Present in SLCC7179 (3a), FSLR2561 and SLCC2372 (1/2c), EGD-e (1/2a), and SLCC 2479 (3c).

f Present in J0161 only.

g Present in 4a strains (M7, L99 and HCC23).

h Present in 10403S only.

Among the 447 genes upregulated in strain H7858 after bile exposure, 33 protein-coding genes had no homologs in strain 10403S (Table 3). These 33 genes included (i) 6 genes present in all lineage I genomes analyzed and (ii) 27 genes that were found in some but not all lineage I strains. No genes were found to be lineage-specific, however, we identified four “serotype-specific” genes found in all serotype 4 [4b, 4d, and 4e] strains, but none of the other lineage I genomes. Interestingly, one of the “serotype 4 specific genes”, peg_17, is part of a three-gene operon and codes for a protein with homology to an N-acetylmuramoyl-L-alanine amidase possibly involved in peptidoglycan synthesis.

**Table 3:** Genes upregulated by bile in H7858 with no homolog in 10403S.

**H7858 Locus (peg_)**	**FC**	**Function**	**Presence in^a^:**						
			**Lineage I**	**Lineage II**	**Lineage III**	***L. innocua***	***L. ivanovii***	***L. seeligeri***	***L. welshimeri***
1532	4.4	N-acetylmuramic acid 6-phosphate etherase	+	–	V^k^	–	+	+	+
2817	2.7	Transcriptional regulator2C GntR family	+	–	V^l^	+	+	+	+
2818	4.2	Possible membrane fusion protein	+	–	V^l^	+	+	+	+
40	5.5	Hypothetical protein	+	V^j^	–	–	+	–	–
2667	4.4	Substrate binding periplasmic protein MalE	+	V^h^	V^n^	–	+	–	+
2686	2.5	Internalin-like protein Lin0295 homolog	+	V^i^	+	+	–	+	–
16	4.6	Hypothetical protein	V^b^	–	–	–	–	–	–
17	5.4	N-acetylmuramoyl-L-alanine amidase2C family	V^b^	–	–	–	–	–	–
18	4.4	Hypothetical protein	V^b^	–	–	–	–	–	–
500	5.3	FIG00775381: hypothetical protein	V^b^	–	–	–	–	–	–
962	4.1	ABC transporter2C ATP-binding protein	V^c^	–	–	–	–	–	–
2562	2.6	Membrane protein	V^b^	–	–	–	+	–	–
2563	3.0	Glycosyltransferase	V^b^	–	–	–	+	–	–
296	3.6	FIG00774814: hypothetical protein	V^b^	–	V^m^	+	–	–	+
297	4.9	Wall-associated protein	V^b^	–	+	+	–	–	+
667	2.9	Reverse transcriptase	V^o^	V^d^	–	–	–	–	–
2114	4.3	Hypothetical protein	V^o^	V^e^	–	+	–	–	–
2139	2.8	Hypothetical protein	V^o^	V^f^	–	–	–	–	–
2140	3.4	Hypothetical protein	V^o^	V^f^	–	–	–	–	–
2151	2.7	Protein gp34 [Bacteriophage A118]	V^o^	V^g^	–	+	–	–	–
2153	2.8	Hypothetical protein	V^o^	V^g^	–	+	–	–	–
2152	3.1	Lin2383 protein	V^o^	V^g^	V^n^	–	–	–	–
2143	4.2	Hypothetical protein	V^o^	–	–	–	–	–	–
2144	2.7	Protein gp38 [Bacteriophage A118]	V^o^	–	–	–	–	–	–
2145	3.6	Transcriptional regulator	V^o^	–	–	–	–	–	–
2147	4.9	Hypothetical protein	V^o^	–	–	–	–	–	–
2895	2.6	Hypothetical protein	V^o^	–	–	–	–	–	–
2896	2.8	Hypothetical protein	V^o^	–	–	–	–	–	–
2897	2.6	Hypothetical protein	V^o^	–	–	–	–	–	–
2898	2.8	Hypothetical protein	V^o^	–	–	–	–	–	–
953	2.8	AAA superfamily ATPase	V^o^	–	–	–	–	–	+
954	2.8	Putative serine protease	V^o^	–	–	–	–	–	+
2127	3.5	SAM:benzoic acid carboxyl methyltransferase	V^o^	–	V^n^	–	–	–	–

a *Gene distribution was characterized among 27 genomes representing L. monocytogenes lineage I (n = 10), lineage II (n = 9), lineage III (n = 4), as well as other Listeria species (L. innocua, L. ivanovii, L. seeligeri, and L. welshimeri; n = 1 each). Genes absent in all genomes searched are labeled as “–”, genes present in all genomes searched are labeled as “+” and genes that showed variable presence are labeled as “V”*.

b *Present in serotype 4b, 4d, and 4e strains. Absent from 3b, 7, and 1/2b strains*.

c *Absent from ATCC 19117 and SLCC2540*.

d *Present in two serotype 3a strains (Finland and SLCC7179). SLCC7179 DNA sequence has only 68% similarity and presents 48 indels in comparison to H7858 sequence*.

e *Strains SLCC2479 (3c), SLCC2372 and FSLR2561 (1/2c), J0161 and 085923 (1/2a)*.

f *Present in strains SLCC2479 (3c) and SLCC2372 (1/2c)*.

g *Present in strains J0161 (1/2a), SLCC2479 (3c), SLCC2372 and FSLR2561 (1/2c)*.

h *Present in J0161 and 085923 (1/2a), SLCC2479 (3c), and SLCC2372 and FSLR2561 (1/2c)*.

i *Present in J0161*.

j *Present in 4 strains (J011, SLCC7179, Finland and 085923)*.

k *Only serotype 4c strain (SLCC2376)*.

l *Present in SLCC2376*.

m *Present in all 4a strains and absent in SLCC2376*.

n *Present in strains 4a (M7, L99, and HCC23)*.

o *Present in H7858 only*.

None of the 187 genes with lower transcript levels in 10403S after bile exposure were classified as absent in H7858. On the other hand, the 249 genes that showed lower transcript levels in the lineage I strain H7858 after bile exposure included 11 protein-coding genes with no homologs in the lineage II strain 10403S (Table 4). These 11 genes represented (i) 10 genes present in all lineage I genomes analyzed and (ii) one gene that was found in some, but not all, lineage I strains. Among the genes present in all lineage I strains, four were absent from all lineage II genomes analyzed, but none were lineage I-specific, as some were present in lineage III strains and other *Listeria* species. Interestingly, one of these genes, peg_2467, codes for a putative internalin 2C that is only present in all lineage I strains analyzed as well as in *L. ivanovii*. Our subsequent analysis found this gene to be SigB-dependent as well (see below). The remaining six genes that were downregulated by bile in H7858 (and present in all lineage I genomes but with no homologs in 10403S), all were present in some, but not all, lineage II and lineage III genomes analyzed. Interestingly, one of these six genes, peg_337, also encodes a putative peptidoglycan bound protein with an LPXTG motif similar to internalins.

**Table 4:** Genes downregulated by bile in H7858 with no homolog in 10403S.

**H7858 Locus (peg_)**	**FC**	**Function**	**Presence in^a^:**						
			**Lineage I**	**Lineage II**	**Lineage III**	***L. innocua***	***L. ivanovii***	***L. seeligeri***	***L. welshimeri***
1531	0.37	PTS system sucrose-specific component	+	–	V^d^	–	–	+	+
526	0.26	Cell wall surface anchor family protein	+	–	–	–	+	+	+
525	0.31	Hypothetical protein	+	–	–	–	–	–	+
2467	0.40	Internalin2C putative (LPXTG motif)	+	–	–	–	+	–	–
338	0.25	Transposase OrfA2C IS3 family	+	V^c^	V^e^	–	–	–	–
337	0.26	Putative peptidoglycan bound protein	+	V^c^	V^e^	–	–	–	–
336	0.29	Hypothetical protein	+	V^c^	V^e^	–	–	–	–
335	0.40	Hypothetical protein	+	V^c^	V^e^	–	–	–	–
334	0.35	Conserved domain protein	+	V^c^	V^e^	–	–	–	–
333	0.36	Conserved domain protein	+	V^c^	V^e^	–	–	–	–
961	0.05	Hypothetical protein	V^b^	–	–	–	–	–	–

a *Gene distribution was characterized among 27 genomes representing L. monocytogenes lineage I (n = 10), lineage II (n = 9), lineage III (n = 4), as well as other Listeria species (L. innocua, L. ivanovii, L. seeligeri, and L. welshimeri; n = 1 each). Genes absent in all genomes searched are labeled as “– “, genes present in all genomes searched are labeled as “+” and genes that showed variable presence are labeled as “V”*.

b *Absent in ATCC (4d) and SLCC2540 (3b)*.

c *Present in EGD-e and J0161 (1/2a), SLCC2479 (3c), SLCC2372 and FSLR2561 (1/2c)*.

d *Present in SLCC2376 (4c)*.

e *Present in 4a strains (M7, L99 and HCC23)*.

### RNA-seq data for 10403s and H7858 Δ*SigB* strains exposed to bile and control conditions reveal new SigB-dependent genes

We found that the two Δ*sigB* strains tested here showed increased sensitivity to bile as compared to their parent strains (see Supplementary Figure 1). We thus characterized the transcriptional response of Δ*sigB* strains for both 10403S and H7858 to bile exposure, using the same conditions described above for the wildtype (WT) strains. Initial analyses were performed to identify genes that showed transcript levels that differed significantly between the respective parent and Δ*sigB* strains (e.g., 10403S WT vs. 10403S Δ*sigB*) in the cells exposed to pH 5.5 (without bile). Overall, 106 and 194 protein coding genes showed significantly higher transcript levels in the 10403S and H7858 parent strains compared to the respective Δ*sigB* strains. In addition, two ncRNAs (*sbrA* and *sbrE*) showed significantly higher transcript levels in both parent strains (Table 5, Supplementary Table 5). When comparisons between parent and Δ*sigB* strains were performed for cells exposed to bile at pH 5.5, a total of 68 protein coding genes and one ncRNA (*sbrA*) showed higher transcript levels in the 10403S parent strain, while 94 protein coding genes and two ncRNAs (*sbrA* and *sbrE*) showed higher transcript levels in the H7858 parent strain (Table 5, Supplementary Table 5). All of the genes identified as SigB-dependent in cells exposed to bile at pH 5.5 were also found as SigB-dependent in cells exposed to only pH 5.5, further supporting that bile exposure does not induce further transcription of the SigB regulon above the induction already caused by pH 5.5 exposure.

**Table 5:** Number of transcripts (protein-coding and non-coding) significantly upregulated in the WT vs Δ*sigB* mutant under control and exposure to bile conditions.

**Condition**	**Strain**	**Upregulated**		
		**Total**	**ncRNAs**	**genes**
Control	10403S	108	2	106
	H7858	196	2	194
Bile	10403S	69	1	68
	H7858	96	2	94

Comparisons with a recently reported SigB pan-regulon (Liu et al., 2017) identified 50 genes that our analyses identified as upregulated by SigB, but that had not previously been reported as SigB-dependent; for 26 of these genes, we identified putative upstream SigB-dependent promoters. These 26 genes include (i) 15 genes that are present in the genomes of both 10403S and H7858, but that met only the FDR and FC threshold in H7858 (Table 6), and (ii) 11 genes that are present in the genome of H7858, but not in 10403S (Table 7). The 15 newly identified SigB-dependent genes found in the genomes of both strains included 1 gene that was characterized by the absence of the putative SigB dependent promoter in 10403S and 14 genes that had putative upstream SigB-dependent promoters in both strains, however the promoters in 10403S showed unique features that could explain a lack of detectable SigB-dependent transcription (Table 6). For example, for 7 genes, the upstream promoter found in strain 10403S displays polymorphisms that may affect recognition by SigB (see Table 6 for details). The 11 newly identified SigB-dependent genes that are present in the genome of H7858, but not in 10403S (Table 7) included (i) 5 genes found in all lineage I genomes, absent from all lineage II genomes, but found in some genomes representing lineage III or other *Listeria* species; and (ii) 6 genes found in all lineage I genomes, some, but not all, genomes representing lineages II and III, but absent from all other *Listeria* spp. genomes.

**Table 6:** Newly identified SigB-dependent genes present in both 10403S and H7858.

**EGDe locus (lmo)**	**Function**	**10403S**				**H7858**			
		**Locus (LMRG_)**	**FDR**	**FC**	**SigB promoter**	**Locus (peg_)**	**FDR**	**FC**	**SigB promoter**
0340	Hypothetical protein	00031	*3.67* × *10^−*1*^*	*1.40*	Yes^i^^,^^ii^	2762	1.89 × 10^−5^	2.75	Yes
0551	Extracellular protein	00233	*1.73* × *10^−*1*^*	*1.61*	Yes^iii^	418	8.12 × 10^−6^	2.62	Yes
0671	Hypothetical protein	00359	*3.80* × *10^−*1*^*	*0.76*	Yes^iii^	539	1.77 × 10^−5^	4.30	Yes
0723	Methyl-accepting chemotaxis protein	00412	*6.23* × *10^−*1*^*	*0.83*	Yes^iii^	591	1.39 × 10^−10^	25.57	Yes
0724	Hypothetical protein	00413	*6.29* × *10^−*1*^*	*0.89*	Yes^iii^	592	1.78 × 10^−11^	17.31	Yes
1300	Arsenic efflux pump protein	00750	6.72 × *10^−*3*^*	*1.81*	Yes^ii^	1164	6.67 × 10^−7^	2.65	Yes
2094	Class II aldolase/adducin domain protein	01245	1.24 × 10^−*2*^	*0.13*	Yes^i^	1957	2.20 × 10^−4^	2.84	Yes
2095	Tagatose-6-phosphate kinase	01246	*7.16* × *10^−*1*^*	*0.44*	Yes^i^	1958	2.11 × 10^−4^	2.78	Yes
2837	Permease protein YcjP	01861	*6.48* × *10^−*1*^*	*1.28*	Yes^i^	2644	1.85 × 10^−6^	4.90	Yes
2836	Zinc-type alcohol dehydrogenase YcjQ	01862	*5.28* × *10^−*1*^*	*1.45*	Yes^i^	2643	4.57 × 10^−6^	4.73	Yes
2835	Sugar phosphate isomerases YcjR	01863	*4.93* × *10^−*1*^*	*1.41*	Yes^i^	2642	2.17 × 10^−5^	3.27	Yes
2834	Putative oxidoreductase YcjS	01864	*3.63* × *10^−*1*^*	*1.67*	Yes^i^	2641	6.71 × 10^−5^	3.88	Yes
0821	Hypothetical protein	02246	*5.52* × *10^−*2*^*	*1.49*	Yes^ii^	679	3.22 × 10^−4^	2.54	Yes
0075	Probable phosphorylmutase	02326	*2.55* × *10^−*1*^*	*1.26*	No	95	7.74 × 10^−6^	2.61	Yes
0105	Chitinase	02354	*5.08* × *10^−*1*^*	*1.39*	Yes^ii^	125	8.57 × 10^−6^	3.33	Yes

*Values in italics indicate those that did not meet the cutoff of FDR < 0.05 or FC > 2.5*.

i *SigB-dependent promoter is polymorphic*.

ii *SigA-dependent promoter masks signal from SigB-dependent promoter*.

iii *SigB promoter present, but additional unidentified regulatory mechanism prevents transcription*.

**Table 7:** SigB-dependent genes in H7858 with no homolog in 10403S.

**H7858 Locus (peg_)**	**Function**	**FC**	**Promoter Sequence**	**Presence in^a^:**						
				**Lineage I**	**Lineage II**	**Lineage III**	***L. innocua***	***L. ivanovii***	***L. seeligeri***	***L. welshimeri***
2467	Internalin2C putative	28.2	a**gttt**(N15)**gggaaa**a	+	–	–	–	+	–	–
525	DNA-binding protein	52.6	c**gatt**(N15)**gggtat**a	+	–	–	–	–	–	+
526	Cell wall surface anchor family protein	28.8	c**gatt**(N15)**gggtat**a	+	–	–	–	+	+	+
1531	PTS system component	20.1	g**gttt**(N17)**gggtaa**t	+	–	V^c^	–	–	+	+
2775	Internalin-like protein	34.5	t**gttt**(N16)**gggaac**a	+	–	+	+	–	–	–
333	Conserved domain protein	9.0	t**gttt**(N15)**gggata**t	+	V^b^	V^d^	–	–	–	–
334	Conserved domain protein	7.9	t**gttt**(N15)**gggata**t	+	V^b^	V^d^	–	–	–	–
335	FIG00774095: hypothetical protein	6.8	c**ctat**(N16)**gtgatt**a	+	V^b^	V^d^	–	–	–	–
336	FIG00775197: hypothetical protein	8.5	c**ctat**(N16)**gtgatt**a	+	V^b^	V^d^	–	–	–	–
337	Putative peptidoglycan bound protein	8.2	c**ctat**(N16)**gtgatt**a	+	V^b^	V^d^	–	–	–	–
338	Transposase OrfA2C IS3 family	9.4	c**ctat**(N16)**gtgatt**a	+	V^b^	V^d^	–	–	–	–

*Bold letters indicate −35 and −10 regions of the promoter*.

a *Gene distribution was characterized among 27 genomes representing L. monocytogenes lineage I (n = 10), lineage II (n = 9), lineage III (n = 4), as well as other Listeria species (L. innocua, L. ivanovii, L. seeligeri and L. welshimeri; n = 1 each). Genes absent in all genomes searched are labeled as “– “, genes present in all genomes searched are labeled as “+” and genes that showed variable presence are labeled as “V”*.

b *Absent in 10403S, SLCC7179, SLCC5850, Finland and 85923*.

c *Only SLCC2376 (strain 4c)*.

d *Absent in SLCC2376*.

While annotation of the 26 newly identified SigB-dependent genes described above classified a number of them as hypothetical proteins, these genes were also found to include (i) a gene classified as encoding a chemotaxis protein, (ii) the *ycjPQRS* operon (LMRG_01861 - LMRG_01864; peg_2641 - peg_2644), which is involved in sugar utilization, and (iii) a number of genes encoding proteins similar to internalins (see Tables 6, 7 for details). One example of a protein similar to internalins is peg_2467, which was also found to be bile responsive, as described above.

## Discussion

Our data show that bile exposure at pH 5.5 elicits a broad response in *L. monocytogenes*, which is characterized by upregulation of motility, cell wall modification mechanisms, and the PrfA regulon. Induction of the bile stimulon hence includes responses that likely facilitate survival during the gastrointestinal stages of infection that follow bile exposure, such as motility, which may enable infection in the small intestine. Furthermore, the fact that bile exposure induces the PrfA regulon, but does not seem to induce further upregulation of the SigB regulon (beyond that expected by exposure to pH 5.5), suggests a model where SigB-dependent gene expression is more important at an earlier stage of gastrointestinal infection (e.g., acid exposure in the stomach) than PrfA-dependent gene expression.

### *L. monocytogenes* induces a large bile stimulon in response to bile stress at pH 5.5, which includes lineage- and strain-specific patterns

RNA-seq analyses of *L. monocytogenes* strains 10403S and H7858 exposed to bile identified 460 and 696 transcripts (including protein coding genes and ncRNAs), respectively, that were significantly differentially transcribed (either up- or downregulated) after bile exposure at pH 5.5. This indicates that bile exposure affects transcription of a large proportion of the *L. monocytogenes* genome (representing ~16% and 23% of the genes identified in the two strains tested). Analyses of the data for both strains uncovered (i) a bile pan-stimulon (defined as genes that showed significantly higher transcript levels after bile exposure in at least one strain) of 504 protein-coding genes and 32 ncRNAs and (ii) a bile core stimulon of 182 protein-coding genes and 2 ncRNAs. This broad and robust response indicates a highly regulated response to an important GI hurdle encountered by *L. monocytogenes*. While a previous proteomic study of *L. monocytogenes* (Payne et al., 2013) identified alterations in levels of proteins associated with DNA repair, chaperone activity, and oxidative stress response in response to bile exposure under anaerobic and neutral pH conditions, it did not detail either a pan or core bile stimulon (Payne et al., 2013). Similarly, a more recent proteomic study also identified a number of genes induced in *L. monocytogenes* in response to bile stress under neutral pH aerobic and anaerobic conditions, but did not detail an overall bile stimulon (Wright et al., 2016). However, a transcriptomic study of the bile regulon for *L. monocytogenes* 10403S, also using bile exposure at neutral pH (Quillin et al., 2011), confirmed a large rearrangement of the transcriptomic landscape after bile exposure with 391 differentially expressed genes after bile exposure; similar to our findings this response was characterized by up-regulation of many cellular processes including cell wall processes and transport related functions.

Our comparative genomic analyses revealed that the bile pan-stimulon of *L. monocytogenes* lineage I and lineage II strains also includes bile responsive, lineage- and strain-specific patterns. For example, we identified six bile responsive, lineage II specific genes that were upregulated by bile exposure in the lineage II strain 10403S and found in the genomes of all other lineage II strains analyzed, but were absent from H7858 and all other lineage I strains analyzed as well as from lineage III strains and other *Listeria* spp. Five of these genes, LMRG_01117—LMRG_01121, are part of a seven gene operon that includes genes encoding an integral membrane protein, a PTS system enzyme and a GntR family transcriptional regulator. Interestingly, in lineage II strains EGD (Joseph et al., 2006) and 10403S (Lobel et al., 2012), these genes were found to be required for and induced by intracellular growth, respectively, and hence may represent lineage specific bile responsive genes that facilitate survival during intestinal stages of infection by facilitating intracellular growth and survival in intestinal epithelial cells. We also identified six genes that were upregulated by bile in lineage I strain H7858 (serotype 4b), but were absent from the genomes of 10403S as well as from all other lineage II strains analyzed; these genes were only present in lineage I strains that were either serotype 4b, 4d, or 4e. These findings are consistent with previous studies that also showed that lineage I serotype 4 strains show distinct gene presence/absence patterns as compared to lineage I serotype 1/2b strains (Lei et al., 2001; Doumith et al., 2004). Interestingly, we also identified three bile responsive genes that were annotated as encoding internalins with all three of these genes present in all lineage I strains analyzed, but absent in all or some lineage II strains, suggesting a possible role in virulence of lineage I strains. Overall, our identification of lineage- and strain-specific bile responsive genes is consistent with a number of studies that have shown phenotypic, genotypic, and ecologic differences between *L. monocytogenes* lineages (Severino et al., 2007; den Bakker et al., 2010; Oliver et al., 2010; Orsi et al., 2011; Tsai et al., 2011). For example, a transcriptomic study comparing strains representing all four lineages of *L. monocytogenes* and their isogenic Δ*sigB* mutants identified a pan-regulon consisting of ~400 genes that are SigB-dependent in at least one strain and a small core regulon consisting of ~60 genes that are SigB-dependent in all of the strains (Oliver et al., 2010). A separate comparative transcriptomic study using six strains representing lineage I and II, grown to late logarithmic phase, identified differences in virulence, cell wall and stress response between the two lineages (Severino et al., 2007). Furthermore, strain differences in bile sensitivity have been documented in *L. monocytogenes* (White et al., 2015) and *Lactobacillus plantarum*, a probiotic and commensal bacterium commonly found in the gastrointestinal tract (Hamon et al., 2011), suggesting that strains and clonal groups within bacterial species may show specific adaptation to the gastrointestinal tract that should be explored to develop a better understanding of enteric pathogen transmission. While our comparative genomic analyses suggest that we have identified lineage-, serotype-, and strain-specific genes conserved in *L. monocytogenes* and other *Listeria* species, RNA-seq experiments on additional strains representing different *L. monocytogenes* lineages will be needed to confirm lineage-specific responses to bile exposure.

### Bile exposure induces transcription of genes encoding motility and cell wall modification functions, which likely facilitates survival and infection of *L. monocytogenes* at the Gi stage of infection

Our data identified a number of gene groups and genes that are induced specifically by bile and that are likely to facilitate infection and survival during the intestinal stages of infection. Interestingly, a number of the flagellar motility genes that were significantly upregulated in H7858, also had higher transcripts in 10403S exposed to bile, but did not meet the cutoff to be considered significant. This finding is consistent with observations that 10403S shows a higher expression of flagellar genes at 37°C (Gründling et al., 2004; Way et al., 2004) as compared to other *L. monocytogenes* strains that downregulate flagella expression at this temperature. Therefore, transcript levels of flagellar-related genes are likely to show fewer differences between 10403S grown in BHI at 37°C with and without bile, due to the higher background transcript levels in 10403S cells grown in BHI at 37°C without bile.

Overall, we identified over 40 motility-related genes (in six operons) that were induced after exposure to bile in both strains tested. The finding that genes coding for motility- and flagella-associated proteins are induced by bile is consistent with several previous studies. For example, a previous proteomic study in *L. monocytogenes* found that expression of flagellin and, for certain strains, flagellar motor proteins is induced by exposure to bile under aerobic conditions (Wright et al., 2016). Additionally, in Gram-negative bacteria, motility has been shown to be responsive to bile exposure in different bacteria, although with somewhat contradictory results; for example, in *E. coli* (de Jesus et al., 2005; Hamner et al., 2013) and *Salmonella* (Prouty et al., 2004), bile exposure leads to a decrease in flagella expression and downregulation of motility, whereas in *Vibrio cholerae* (Gupta and Chowdhury, 1997; Schuhmacher and Klose, 1999; Chatterjee et al., 2007) and *Campylobacter jejuni* (Allen and Griffiths, 2001; Fox et al., 2007; Kreuder et al., 2017), exposure to bile has been shown to induce flagella expression and motility. Functionally, flagella have been found to be important for invasion of intestinal epithelial cells and the intestinal stages of infection in some bacterial pathogens (Haiko and Westerlund-Wikstrom, 2013), including studies that show the importance of flagella for invasion of epithelial cells in *L. monocytogenes* (Dons et al., 2004; Bigot et al., 2005). Specifically, it has been shown that motile *L. monocytogenes* cells outcompete non-motile cells in colonization of the intestine after infection (O'Neil and Marquis, 2006). In summary, even though previous studies have shown that motility in *L. monocytogenes* is typically downregulated at 37°C, presumably to avoid host recognition (Dons et al., 2004; Way et al., 2004), there is considerable evidence that induction of flagella expression under specific conditions (e.g., in the intestinal tract) may be important for infection. Hence, it seems plausible that, after passage into the duodenum, bile serves as a signal for *L. monocytogenes* to up-regulate flagellar expression before reaching the epithelium.

Another characteristic of the bile stimulon was the induction of genes with cell wall modification related functions. Cell envelope modification mechanisms are known strategies bacteria use to survive under rapidly changing environmental conditions (Carvalho et al., 2014). Our RNA-seq data showed the *dltABCD* operon is induced under bile exposure for both strains 10403S and H7858, consistent with other studies where *dltA* was also found to be upregulated under bile exposure in *Lactobacillus plantarum* (Bron et al., 2006) and *Lactobacillus rhamnosus* (Koskenniemi et al., 2011). The DltABCD proteins are involved in the D-alanylation of teichoic acids (Perego et al., 1995), which facilitates resistance against antimicrobial peptides (Revilla-Guarinos et al., 2014). D-alanylation of teichoic acids by the Dlt proteins have been shown to play a role in resistance to lysozyme (Guariglia-Oropeza and Helmann, 2011), nisin (Kingston et al., 2013; Kang et al., 2015) and other cell wall acting stresses (McBride and Sonenshein, 2011) in Gram positive bacteria. While bile resistance has not been linked to D-alanylation of teichoic acids, it seems plausible that such modification of the cell envelope could affect the interaction with bile in a protective manner. Several other cell envelope modification mechanisms were found here to be induced after bile exposure, including Mg transport functions. Increasing concentrations of Mg have been shown to be able to rescue cell wall defects (Domínguez-Cuevas et al., 2013), restore normal growth of cell wall deficient mutants (Formstone and Errington, 2005), and spheroid perpetuation of cell division deficient mutants (Leaver and Errington, 2005), suggesting an important role for Mg transport in cell wall homeostasis. Hence it would seem feasible that increased Mg transport after bile exposure may enhance *L. monocytogenes* survival of bile or other membrane-related stresses encountered in the intestinal tract after bile exposure.

### Bile exposure of *L. monocytogenes* induces upregulation of the PrfA regulon and downregulation of the SigB regulon, supporting a model where SigB-dependent gene expression plays an important role at the earlier stages of gastrointestinal infection

Since SigB and PrfA are key regulators of gene expression in *L. monocytogenes*, with both contributing to regulation of virulence genes, we hypothesized that expression of their regulons would be affected by bile exposure. Additionally, we found that the two Δ*sigB* strains tested here showed increased sensitivity to bile as compared to their parent strains (see Supplementary Figure 1), which is consistent with previous studies (Sue et al., 2003; Zhang et al., 2011) that have identified a role for SigB in resistance to bile. Surprisingly, our data indicate that bile exposure at pH 5.5 upregulates a large part of the PrfA regulon, but does not upregulate expression of the SigB regulon above the level of induction caused by exposure to acidic pH. In fact, bile exposure may actually downregulate at least some components of the SigB regulon. In contrast to our findings, a previous study reported repression of the PrfA regulon in response to bile exposure (Quillin et al., 2011). This previous study also identified BrtA (previously TetR) as induced by bile exposure; however this gene did not show increased transcript levels under bile exposure here. However, bile exposure in this previous study (Quillin et al., 2011) was performed at neutral pH, as compared to bile exposure at pH 5.5 performed here. Since *L. monocytogenes* is exposed to bile as it enters the duodenum (gradient from pH 2 to pH 5) (Ovesen et al., 1986), we believe our experimental set up represents a more physiologically-relevant condition. Interestingly, we found that several members of the PrfA regulon, such as *hly, actA, plcA*, and *plcB*, were significantly upregulated after exposure to bile in both strains, while *inlA* and *inlB*, which previously had been reported as co-regulated by SigB and PrfA, were not upregulated in response to bile. PrfA-regulated genes that are co-regulated by SigB were not upregulated in response to bile, presumably due to the fact that our control condition already induced SigB. In particular, we found that transcription of *bilEA* and *bsh*, which have been previously reported to be involved in bile resistance (Begley et al., 2005; Sleator et al., 2005) and are also co-regulated by SigB and PrfA (Sleator et al., 2005; Zhang et al., 2011), was not induced by bile exposure at pH 5.5 in the WT strains but were confirmed as SigB-dependent under exposure to pH 5.5 with and without bile. Interestingly, *pva* and *btlB*, two other genes that were previously described as contributing to bile resistance (with no evidence for SigB-dependent regulation) (Begley et al., 2005), were also not found to be induced by bile here, which is consistent with previous studies (Quillin et al., 2011; Payne et al., 2013; Wright et al., 2016) that have also not reported these genes to be induced by bile.

The finding that bile exposure induces the PrfA regulon, with no further upregulation of the SigB regulon (beyond that expected by exposure to pH 5.5), supports a model where SigB-dependent gene expression may play a more important role at the earlier stages of gastrointestinal infection (e.g., acid exposure in the stomach) than PrfA-dependent gene expression, while conditions encountered upon transition into the small intestine (i.e., bile exposure) induce PrfA, which may prime *L. monocytogenes* for intracellular infections (e.g., of intestinal epithelial cells) that are initiated in the small intestine. The observation that key genes that are likely important immediately after transition from the stomach to the small intestine (e.g., *inlA, bsh*) are co-regulated by SigB, but are not further induced by bile stress, also suggests that acid conditions such as those encountered in the stomach prime *L. monocytogenes* for survival in the small intestine, where transcription of genes that are co-regulated by PrfA may be maintained (but not increased) through bile mediated induction of the PrfA regulon. Hence survival through the gastrointestinal tract appears to be facilitated by a relay of reactive and anticipatory transcriptional regulation mechanisms.

Our data indicate that a large proportion of the SigB regulon, including some lineage specific SigB-dependent genes (e.g., peg_2467, which encodes a putative internalin protein) is downregulated under bile exposure. This is surprising since SigB has been linked to resistance and tolerance to bile in *L. monocytogenes* (Kazmierczak et al., 2003; Zhang et al., 2011). The most likely explanation for our finding that at least part of the SigB regulon is downregulated under bile exposure is the fact that we characterized the bile stimulon by comparing transcriptional profiles of strains exposed to bile at pH 5.5 to strains exposed to pH 5.5 without bile; these types of acidic conditions are expected to induce expression of the SigB regulon (Sue et al., 2004; Ivy et al., 2012). Interestingly, we found that oxidation-reduction processes were downregulated after bile exposure, including through SigB-independent mechanisms. This downregulation could be beneficial for *L. monocytogenes* as exposure to bile typically is a signal for transition into the reduced oxygen tension environment of the small intestine (He et al., 1999; Espey, 2013), where expression of oxidative stress response systems is not likely to be beneficial for *L. monocytogenes*. This is also consistent with other studies that have suggested that transition to anaerobic and oxygen restricted environments enhance subsequent infectivity of *L. monocytogenes* (Bo Andersen et al., 2007).

### Sequential environmental cues in the gastrointestinal environment provides signals for a relay of reactive and anticipatory transcriptional regulation mechanisms

Overall, our data support an emerging model where transcriptional responses to environmental cues and stresses not only facilitate survival of a specific stress after exposure to a given stress is initiated, but also provide signaling cues that facilitate anticipatory responses to subsequent stress conditions. We propose that these types of regulatory mechanisms are particularly important during infection of homeothermic hosts and specifically the gastrointestinal stages of infection, where the sequence of environmental cues encountered is highly standardized and reproducible. This hypothesis is supported by prior work on *Vibrio cholerae*, which suggested that mucosal penetration primes bacterial cells for subsequent host colonization (Liu et al., 2008). Specifically, our data support that SigB-dependent gene expression in *L. monocytogenes* plays an important role at early stages of gastrointestinal infection where it not only facilitates survival of acid exposure in the stomach, but also primes *L. monocytogenes* for survival and growth in subsequently encountered sites (e.g., through induction of bile resistance mechanisms and internalin A expression). Subsequently, bile exposure at acidic pH represents a signal that induces a substantial re-shaping of the transcriptome, which includes initiation of expression of the PrfA regulon as well as other functions important for growth and survival in the small intestine, which is encountered after bile released into the duodenum, the first portion of the small intestine.

## Data availability

RNA-seq data are accessible through GEO Series accession number GSE103443. Additional datasets and the R code used for Bayseq and Goseq analyses are accessible on GitHub (https://github.com/renatorsi/Guariglia-Oropeza2017BileStimulonL_monocytogenes).

## Author contributions

VG-O performed the RNA-seq experiments and data analyses; RO performed the comparative genomics analyses; VG-O, CG, MW, and KB co-wrote the manuscript and conceived the study. All authors read and approved the final manuscript.

### Conflict of interest statement

The authors declare that the research was conducted in the absence of any commercial or financial relationships that could be construed as a potential conflict of interest.

## Supplementary material

Supplementary Figure 1 Bile exposure effect on strains 10403S and H7858.

Supplementary Table 1 Combined BaySeq analysis for 10403S and H7858 with and without exposure to bile.

Supplementary Table 2 Enriched GO terms in genes upregulated by bile in 10403S and H7858.

Supplementary Table 3 Examples of functionally interesting genes upregulated by bile.

Supplementary Table 4 Enriched GO terms in genes downregulated by bile in 10403S and H7858.

Supplementary Table 5 Combined BaySeq analysis for 10403S and H7858 comparing parent and Δ*sigB* mutant, with and without exposure to bile.
