# Supplementary figures and images for: The Listeria monocytogenes Bile Stimulon under Acidic Conditions Is Characterized by Strain-Specific Patterns and the Upregulation of Motility, Cell Wall Modification Functions, and the PrfA Regulon
Source: Front Microbiol. 2018 Feb 6;9:120. doi: 10.3389/fmicb.2018.00120 (PMC5808219; doi:10.3389/fmicb.2018.00120)

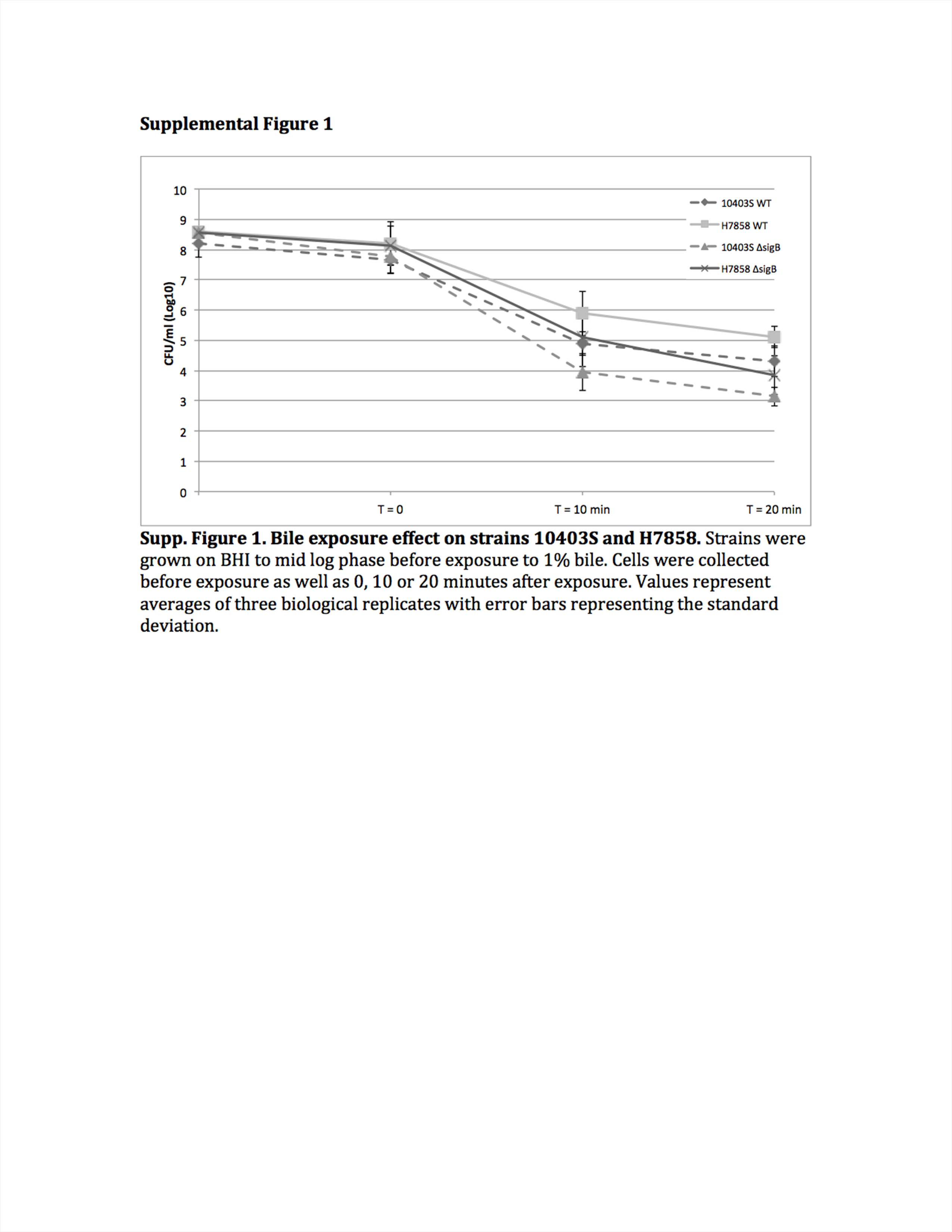

Supplement: Supplementary Figure 1 — Bile exposure effect on strains 10403S and H7858. [file Image1.TIFF]
