# Supplementary material for: The Listeria monocytogenes Bile Stimulon under Acidic Conditions Is Characterized by Strain-Specific Patterns and the Upregulation of Motility, Cell Wall Modification Functions, and the PrfA Regulon
Source: Front Microbiol. 2018 Feb 6;9:120. doi: 10.3389/fmicb.2018.00120 (PMC5808219; doi:10.3389/fmicb.2018.00120)
Supplement: Supplementary Table 2 — Enriched GO terms in genes upregulated by bile in 10403S and H7858. [file Table2.DOCX]

**Supp. Table 2.** Enriched GO terms in genes upregulated by bile in 10403S and H7858

|  |  | Significantly enriched in: | |
| --- | --- | --- | --- |
| GO Term (GO:) | Annotation | 10403S | H7858 |
| 0007165 | signal transduction | + | + |
| 0009401 | phosphoenolpyruvate-dependent sugar phosphotransferase system | + | + |
| 0007154 | cell communication | + | + |
| 0008643 | carbohydrate transport | + | + |
| 0005215 | transporter activity | + | + |
| 0015144 | carbohydrate transmembrane transporter activity | + | + |
| 0015846 | polyamine transport | + | - |
| 0048870 | cell motility | + | - |
| 0015847 | putrescine transport | + | - |
| 0015848 | spermidine transport | + | - |
| 0008982 | protein-N(PI)-phosphohistidine-sugar phosphotransferase activity | + | - |
| 0006810 | transport | - | + |
| 0051234 | establishment of localization | - | + |
| 0008982 | protein-N(PI)-phosphohistidine-sugar phosphotransferase activity | - | + |
| 0006935 | chemotaxis | - | + |
| 0007610 | behavior | - | + |
| 0007626 | locomotory behavior | - | + |
| 0040011 | locomotion | - | + |
| 0042330 | taxis | - | + |
| 0034219 | carbohydrate transmembrane transport | - | + |
| 0009357 | protein-N(PI)-phosphohistidine-sugar phosphotransferase complex | - | + |
| 0022857 | transmembrane transporter activity | - | + |
| 0022892 | substrate-specific transporter activity | - | + |
| 0016773 | phosphotransferase activity, alcohol group as acceptor | - | + |
| 0022891 | substrate-specific transmembrane transporter activity | - | + |
| 0022804 | active transmembrane transporter activity | - | + |
| 0015424 | amino acid-transporting ATPase activity | - | + |
| 0005351 | sugar:proton symporter activity | - | + |
| 0005402 | cation:sugar symporter activity | - | + |
| 0015295 | solute:proton symporter activity | - | + |
| 0015294 | solute:cation symporter activity | - | + |
| 0015293 | symporter activity | - | + |
| 0051119 | sugar transmembrane transporter activity | - | + |
| 0015291 | secondary active transmembrane transporter activity | - | + |
| 0016740 | transferase activity | - | + |
| 0055085 | transmembrane transport | - | + |
| 0005975 | carbohydrate metabolic process | - | + |
| 0005996 | monosaccharide metabolic process | - | + |
| 0008324 | cation transmembrane transporter activity | - | + |
| 0050794 | regulation of cellular process | - | + |
| 0050789 | regulation of biological process | - | + |
| 0044262 | cellular carbohydrate metabolic process | - | + |
| 0019318 | hexose metabolic process | - | + |
| 0065007 | biological regulation | - | + |
| 0016772 | transferase activity, transferring phosphorus-containing groups | - | + |
| 0015075 | ion transmembrane transporter activity | - | + |
| 0006098 | pentose-phosphate shunt | - | + |
| 0006740 | NADPH regeneration | - | + |
| 0008645 | hexose transport | - | + |
| 0006739 | NADP metabolic process | - | + |
| 0005353 | fructose transmembrane transporter activity | - | + |
| 0015749 | monosaccharide transport | - | + |
