# Supplementary material for: The Listeria monocytogenes Bile Stimulon under Acidic Conditions Is Characterized by Strain-Specific Patterns and the Upregulation of Motility, Cell Wall Modification Functions, and the PrfA Regulon
Source: Front Microbiol. 2018 Feb 6;9:120. doi: 10.3389/fmicb.2018.00120 (PMC5808219; doi:10.3389/fmicb.2018.00120)
Supplement: Supplementary Table 4 — Enriched GO terms in genes downregulated by bile in 10403S and H7858. [file Table4.DOCX]

**Supp. Table 4.** Enriched GO terms in genes downregulated by bile in 10403S and H7858.

|  |  | Significantly enriched in: | | | |
| --- | --- | --- | --- | --- | --- |
| GO Term (GO:) | Annotation | 10403SWT | H7858 WT | 10403S Δ*sigB* | H7858 Δ*sigB* |
| 0006828 | manganese ion transport | + | + | + | + |
| 0016491 | oxidoreductase activity | + | + | + | + |
| 0055114 | oxidation-reduction process | + | + | + | + |
| 0016209 | antioxidant activity | + | + | + | - |
| 0016668 | oxidoreductase activity, acting on a sulfur group of donors, NAD(P) as acceptor | + | + | + | - |
| 0015418^a^ | quaternary-ammonium-compound-transporting ATPase activity | + | + | - | - |
| 0015695^a^ | organic cation transport | + | + | - | - |
| 0015697^a^ | quaternary ammonium group transport | + | + | - | - |
| 0030104^a^ | water homeostasis | + | + | - | - |
| 0042592 | homeostatic process | + | + | - | - |
| 0015990 | electron transport coupled proton transport | + | - | + | + |
| 0016679 | oxidoreductase activity, acting on diphenols and related substances as donors | + | - | + | + |
| 0016682 | oxidoreductase activity, acting on diphenols and related substances as donors, oxygen as acceptor | + | - | + | + |
| 0004791 | thioredoxin-disulfide reductase activity | + | - | + | - |
| 0015226 | carnitine transmembrane transporter activity | + | - | + | - |
| 0004601 | peroxidase activity | + | - | - | - |
| 0015837 | amine transport | + | - | - | - |
| 0015838 | amino-acid betaine transport | + | - | - | - |
| 0016684 | oxidoreductase activity, acting on peroxide as acceptor | + | - | - | - |
| 0019430 | removal of superoxide radicals | + | - | - | - |
| 0031460 | glycine betaine transport | + | - | - | - |
| 0048878^a^ | chemical homeostasis | + | - | - | - |
| 0050662 | coenzyme binding | - | + | + | - |
| 0046655 | folic acid metabolic process | - | + | - | - |
| 0046656 | folic acid biosynthetic process | - | + | - | - |
| 0004129 | cytochrome-c oxidase activity | - | - | + | + |
| 0006091^a^ | generation of precursor metabolites and energy | - | - | + | + |
| 0006123 | mitochondrial electron transport, cytochrome c to oxygen | - | - | + | + |
| 0006812 | cation transport | - | - | + | + |
| 0015002 | heme-copper terminal oxidase activity | - | - | + | + |
| 0016675 | oxidoreductase activity, acting on a heme group of donors | - | - | + | + |
| 0016676 | oxidoreductase activity, acting on a heme group of donors, oxygen as acceptor | - | - | + | + |
| 0022900 | electron transport chain | - | - | + | + |
| 0022904 | respiratory electron transport chain | - | - | + | + |
| 0045277 | respiratory chain complex IV | - | - | + | + |
| 0006970 | response to osmotic stress | - | - | + | - |
| 0016667 | oxidoreductase activity, acting on a sulfur group of donors | - | - | + | - |
| 0051184 | cofactor transporter activity | - | - | + | - |

^a^GO terms previously found enriched in SigB-regulated genes (Y. Liu, R.H. Orsi, K. J. Boor, M. Wiedmann and V. Guariglia-Oropeza, submitted for publication).
